# Osteoid osteoma: the great mimicker

**DOI:** 10.1186/s13244-021-00978-8

**Published:** 2021-03-08

**Authors:** Bruno C. Carneiro, Isabela A. N. Da Cruz, Alípio G. Ormond Filho, Igor P. Silva, Júlio B. Guimarães, Flávio D. Silva, Marcelo A. C. Nico, Xavier M. G. R. G. Stump

**Affiliations:** Department of Musculoskeletal Radiology, Fleury Medicina e Saúde Higienópolis, Rua Mato Grosso 306, 1st Floor, Higienópolis, São Paulo, SP 01239-040 Brazil

**Keywords:** Bone neoplasms, Osteoma, Osteoid, Diagnosis, Differential, Magnetic resonance imaging, Tomography, X-ray computed

## Abstract

Osteoid osteoma is a painful, benign and common bone tumor that is prevalent in young adults. The typical clinical presentation consists of pain that becomes worse at night and is relieved by nonsteroidal anti-inflammatory drugs. The most common imaging finding is a lytic lesion, known as a nidus, with variable intralesional mineralization, accompanied by bone sclerosis, cortical thickening and surrounding bone marrow edema, as well as marked enhancement with intravenous contrast injection. When the lesion is located in typical locations (intracortical bone and the diaphyses of long bones), both characteristic clinical and radiological features are diagnostic. However, osteoid osteoma is a multifaceted pathology that can have unusual presentations, such as intraarticular osteoid osteoma, epiphyseal location, lesions at the extremities and multicentric nidi, and frequently present atypical clinical and radiological manifestations. In addition, many conditions may mimic osteoid osteoma and vice versa, leading to misdiagnosis. Therefore, it is essential to understand these musculoskeletal diseases and their imaging findings to increase diagnostic accuracy, enable early treatment and prevent poor prognosis.

## Key points

- Osteoid osteoma (OO) is a painful, benign and common bone tumor.
- Characteristic clinical and radiological findings are diagnostic, especially for lesions in typical locations.
- Some OO cases present atypical location and unusual imaging findings that can lead to misdiagnosis.
- Many musculoskeletal conditions may present clinical and/or radiological features that mimic OO.

## Background

Osteoid osteoma (OO) was first reported by Jaffe in 1935 [1] in a series of five cases; it is a painful, benign and common tumor, accounting for 3% of all bone neoplasms and 10–12% of benign lesions [2–5]. It is particularly prevalent in Caucasian male adolescents and young adults; moreover, 50% of these tumors occur during the second decade of life, and they rarely occur before the age of 5 and after the age of 35 [3–7].

OO consists of a core called the nidus (the tumor itself) that is typically small, measuring as large as 1.0–2.0 cm and is usually surrounded by corticoperiosteal thickening [1, 2]. Histologically, the nidus comprises an osteoid matrix with variable mineralization, osteoblasts and some osteoclast-type multinucleated giant cells interspersed by a loose fibrovascular stroma, with inflammatory changes and reactional bone formation around the lesion [8].

The typical clinical picture includes intermittent pain that becomes worse at night and is relieved by salicylates [4, 5]. These tumors are highly vascularized and innervated [8], and the physiopathology of pain seems to be related to high levels of prostaglandins (100–1000 × higher than normal), especially prostaglandin E2, increasing the pressure in an innervated bone area within the nidus, particularly in the reactive zone [4, 5, 9–12]. These prostaglandins are also responsible for vasodilatation and edema formation in the surrounding bone marrow and soft tissues [9].

OO most often involves the diaphysis, followed by the metaphysis of the long bones (around 50% and 40%, respectively) [13]. The femur and tibia are involved in more than 50% of cases, and the humerus can also be involved (around 8%) [8, 13]. The spine, hands and feet are involved in approximately 30% of cases; OO more rarely occurs in the skull, scapula, pelvis, ribs, mandible and patella [14, 15]. The spine is involved in approximately 15% of cases [8], and the lumbar spine is the most affected segment of the spine, highlighting that posterior elements are involved in 90% of these cases [16].

Following the radiography-based classification system proposed by Edeiken [17], OO cases can be classified as cortical, cancellous (or medullary) or subperiosteal according to the distribution of the tumor in the axial plane [2]. Cortical OO accounts for the majority of cases (75%), while the cancellous OO accounts for approximately 20% of cases and usually occurs in atypical locations [4, 18]. Subperiosteal OO is the least common type, accounting for as few as 5% of cases [14, 18]. Kayser [19] later proposed a classification system including four types based on sectional studies, subperiosteal, intracortical, endosteal and medullary OO, and hypothesized that all OO cases arise in the subperiosteal area and eventually migrate internally.

**Fig. 1:**
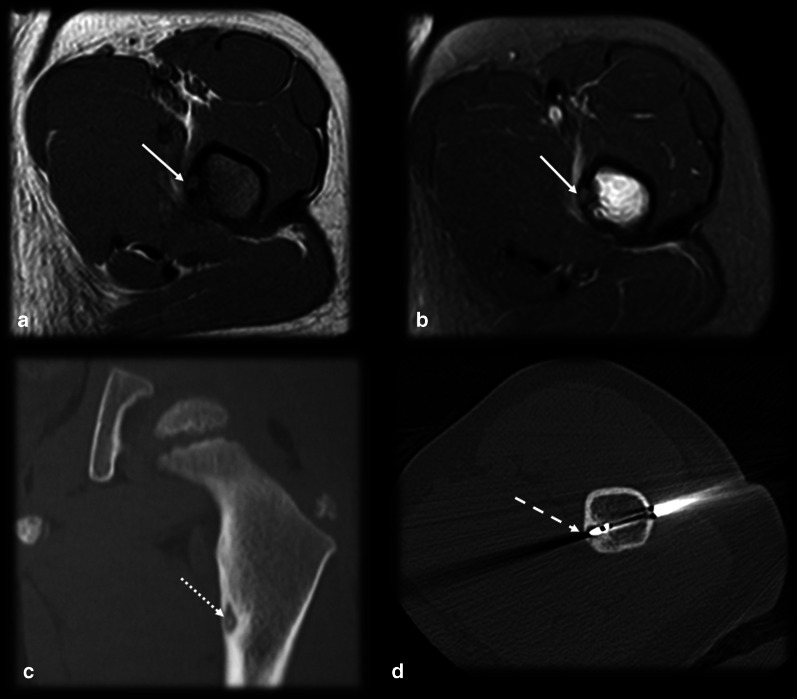
Typical osteoid osteoma and percutaneous ablation. Male, 15 years old, presenting with medial hip pain for 2 months, which became worse at night. Axial T1 (**a**) and T2 FS (**b**) MR images showing a small cortical nidus (arrows) within the femur shaft with a target-like appearance, edema and sclerosis. CT (**c**) better detected the partially mineralized nidus (dotted arrow), cortical thickening and sclerosis than did MRI. CT percutaneous biopsy and radiofrequency ablation (**d**) were performed (dashed arrow)

OO is diagnosed by the combination of both typical clinical picture and imaging findings. Biopsy is recommended at the time of the percutaneous treatment, especially for lesions with atypical presentation, even though it can be nondiagnostic in approximately one-third of cases [20–22]. OO has a natural history of spontaneous regression within 6–15 years, but this period can be reduced to 2–3 years with the use of nonsteroidal anti-inflammatory drugs [5]. Even though pharmacological treatment is an option, due to the adverse effects of the prolonged use of these medications, such as bleeding complications and gastric and renal toxicity, it is reserved for exceptional situations only [23, 24]. The more commonly used treatment options include surgical resection, which is associated with a high morbidity and long recovery period, and percutaneous imaging guided treatments, especially radiofrequency and laser therapy, which have a clinical success rate greater than 90% [21, 22, 25–27] (Fig. 1).

### Diagnostic imaging: typical imaging findings

Conventional radiography (CR) is the usual first-line imaging method used for osteoarticular pain, especially when OO is suspected. The typical radiographic features of OO consist of an intracortical lytic lesion, usually smaller than 1.0 cm, with variable central nidus mineralization associated with reactive surrounding sclerosis and fusiform cortical thickening; the two latter conditions are usually more marked in the pediatric population [4, 5, 14, 16]. OO is typically located at the diaphysis of long bones, and locoregional osteopenia, secondary to pain-related disuse, may occur [4, 16]. The nidus can be distinguished in 85% of cases, and a central area of calcification is identified in 25–50% of cases [2, 4, 8].

Some types of OO are harder to identify on X-rays, such as intraarticular and medullary OO, due to there being less marked corticoperiosteal reactions and spinal OO manifestations because of the complex anatomy and overlapping structures at the spine [4, 14]. In addition, lesions that affect the extremities are even smaller than usual, making the identification of the nidus challenging. When conventional radiographs are not sufficient, other imaging techniques should be used. Even when there is high suspicion of OO on the basis of radiographic and clinical features, sectional imaging studies are performed to better visualize the lesion, confirm the diagnosis and eventually determine the treatment.

CT is considered the modality of choice for OO, as the nidus can be obscured on radiographs. The central calcification may be punctate, amorphous or ring-like, and it is usually regular and centrally located. On CT scans, a “vascular groove” or “CT vessel” sign can be identified, represented by low-density grooves entering the nidus and corresponding to the enlarged vessels that arise from the periosteum to irrigate the hypervascular nidus [9, 28].

The OO nidus shows variable signal intensities on MRI scans with a target-like appearance since nonmineralized vascular stromata have an intermediate/high signal intensity on T2WIs and usually presents intense gadolinium enhancement, while the mineralized portion presents a low signal intensity on all sequences and does not enhance [29, 30]. Surrounding sclerosis and/or inflammatory changes may be abundant and obscure the nidus, making diagnosis difficult [14, 16]. However, the presence of bone marrow edema may help locate the nidus, serving as a red flag and suggesting a more thorough evaluation be conducted in the area of the tumor. Edema is also useful for distinguishing OO from other pathologies that do not promote marked inflammatory changes [30].

Although many studies have suggested that the accuracy of conventional MRI in diagnosing OO is lower than that of CT [14, 31–33], the spatial resolution of modern equipment has improved, volumetric isotropic sequences are now used, and radiologists have become more knowledgeable, so OO can be easily suspected. Evaluations with a small field of view on the axial plane and proton density sequences are preferable [31]. Thus, MRI might be preferred to CT, especially in the pediatric population, to prevent exposure to ionizing radiation.

**Fig. 2:**
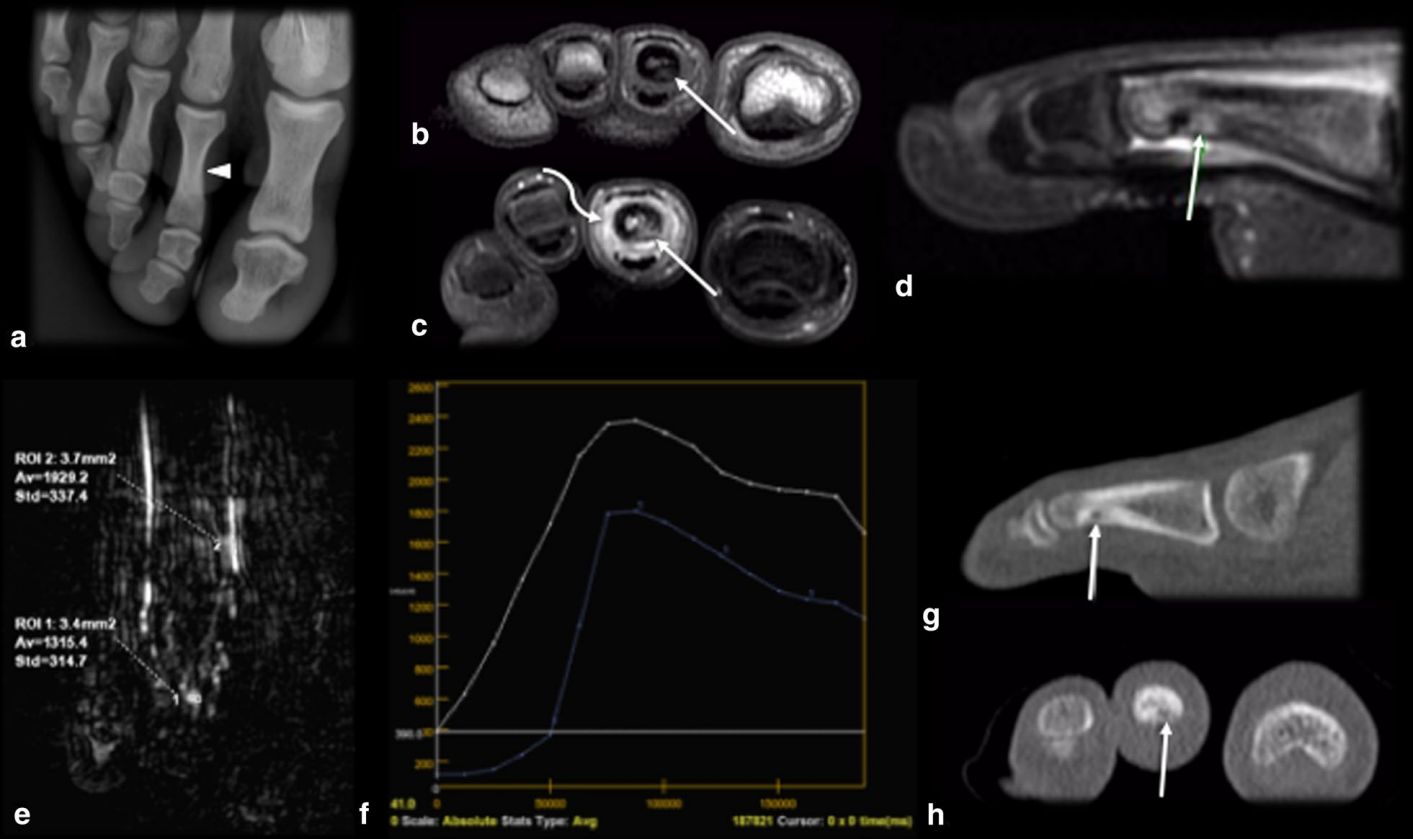
Pattern of enhancement of osteoid osteoma. Male, 17 years old, presenting with metatarsalgia of the right foot for 2 months, which became worse over the past week. He had no recollection of trauma and practiced sports regularly. CR (**a**) showed no significant findings, apart from mild bone sclerosis in the middle phalanx of the second toe (arrowhead). Coronal T1 and T2WI (**b**,** c**) depicted marked edema of the bone marrow and surrounding tissues (curved arrow) and a very small intracortical lesion (dashed arrow). Dynamic MR angiography (**d**–**f**) showed marked enhancement of the intracortical nodule (arrow in **d**), presenting contrast kinetics similar to those of adjacent arteries, with a peak enhancement followed by rapid washout, suggestive of OO (**e**, **f**). A CT scan (**g**,** h**) was later performed, and the findings confirmed the presence of a nidus (arrows)

The use of intravenous contrast may be helpful since the nidus presents strong enhancement due to its prominent vascularity [16]. However, OO enhances with a timing and degree of enhancement similar to those of perilesional arteries, with loss of conspicuity in delayed phases of contrast-enhanced imaging due to progressive perilesional enhancement and rapid washout within the tumor [33]. Therefore, dynamic-contrast images are advantageous to better depict the nidus in early phases of enhancement, presenting a typical curve with rapid inflow followed by washout (curve type IV), typically seen in hypervascular tumors, or less frequently, a peak enhancement followed by a plateau (curve type III) [25, 29, 33] (Fig. 2). Dynamic-contrast studies are able to identify this pattern and may be used in doubtful cases, especially with MRI, since CT has lower contrast resolution between the enhancement and the background bone and exposes the patient to radiation [33]. This method is also useful for detecting residual or recurrent nidus after percutaneous treatment, when the typical imaging features are no longer present, as a sensitivity and specificity greater than 90% have been reported [25].

Bone scintigraphy with technetium-99 has been proven valuable for detecting OO, with a sensitivity of up to 100% [16, 34]. The lesion is usually represented by a central nidus with very high uptake surrounded by a larger area with moderate activity, consisting on the double-density sign, a classic and specific scintigraphic finding of OO [16, 34]. Single-photon emission computed tomography (SPECT) imaging presents higher spatial resolution, specificity and accuracy and allows the detection of smaller lesions when compared to planar scintigraphy [34]. 18F-Labeled sodium fluoride (18F-NaF) PET/CT is also useful for diagnosing OO due to the very intense uptake of this radiotracer within the nidus and sometimes at the perilesional area. Some OO nidi are also FDG-avid and can be identified on FDG-PET/CT scans, with variable intensity [34].

### Atypical imaging findings

**Fig. 3:**
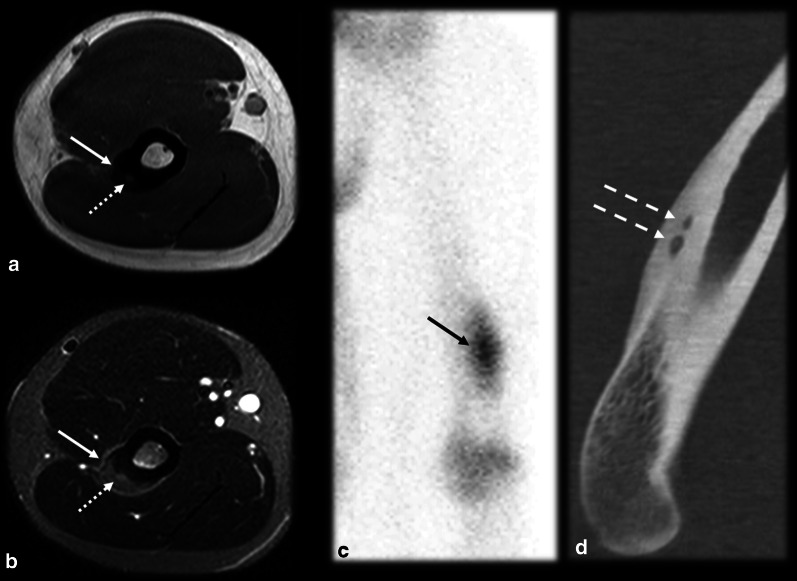
Multicentric osteoid osteoma. A 26-year-old male handball player with lateral elbow and arm pain for 3 months. Orthopedists suspected lateral epicondylitis or a stress reaction. MRI T1 (**a**) and T2 FS (**b**) showed cortical thickening on the lateral supracondylar crest with corticoperiosteal edema (arrow) and small foci of intermediate signal intensity (dotted arrow), which raised the suspicion for OO. Scintigraphy (**c**) evidenced the double density sign (black arrow), which cannot be used to distinguish between single and multicentric OO. CT (**d**) detected two nidi (dashed arrows), confirming the diagnosis of multicentric OO

The typical imaging and clinical findings are diagnostic. However, some OO cases may present with atypical features, which may lead to incorrect diagnoses (Table 1). One type of OO with atypical presentations is multicentric OO. It is a rare condition that is sometimes overlooked and defined as the presence of more than one nidus in the same bone (multicentric, as shown in Fig. 3) or different bones (metachronous), which can cause diagnostic and therapeutic difficulty since all nidi need to be detected and treated. Most often, the nidi are close to each other [4, 35–37].

**Table 1:** OO’s typical and atypical imaging findings

	Typical	Atypical
Number	Single nidus	Multicentric or metachronous
Location within bone	Cortical	Medullary or subperiosteal
Location along bone	Diaphysis	Metaphysis or epiphysis (including intra-articular)
Distribution	Long bones (especially femur and tibia)	Extremities and axial skeleton

**Fig. 4:**
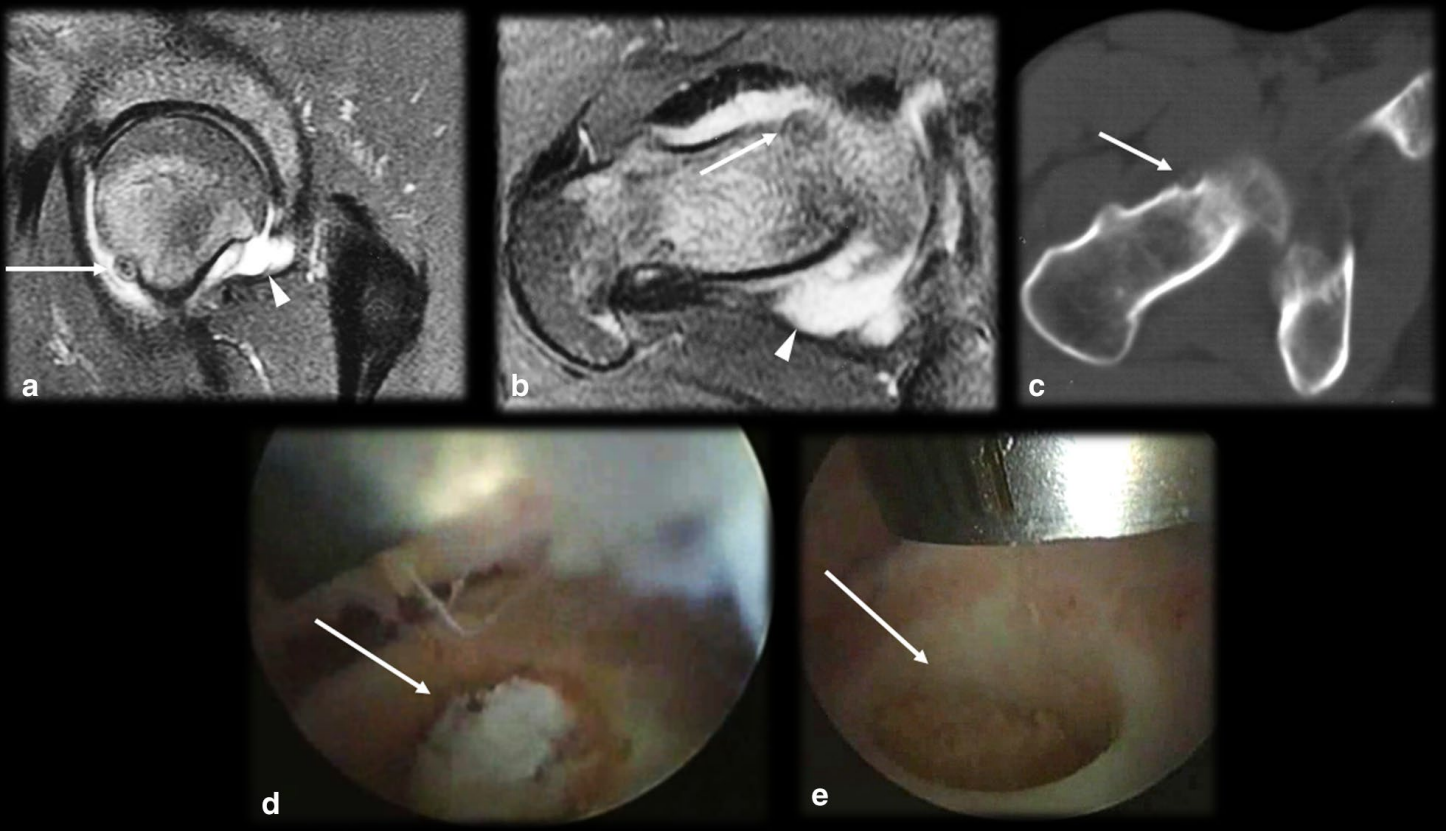
Osteoid osteoma mimicking synovitis of the hip. A 24-year-old man with right hip pain, swelling and tenderness for 2 weeks. Inflammatory marker levels were also elevated. Orthopedists suspected inflammatory arthropathy, and ultrasound (not shown) depicted joint effusion and synovitis. MRI T2 FS sagittal (**a**) and axial (**b**) shows joint effusion (arrowhead) with synovial thickening and a doubtful nidus (arrow), which was better characterized on the CT scan (**c**). Arthroscopic aspect before (**d**) and after (**e**) nidus resection

**Fig. 5:**
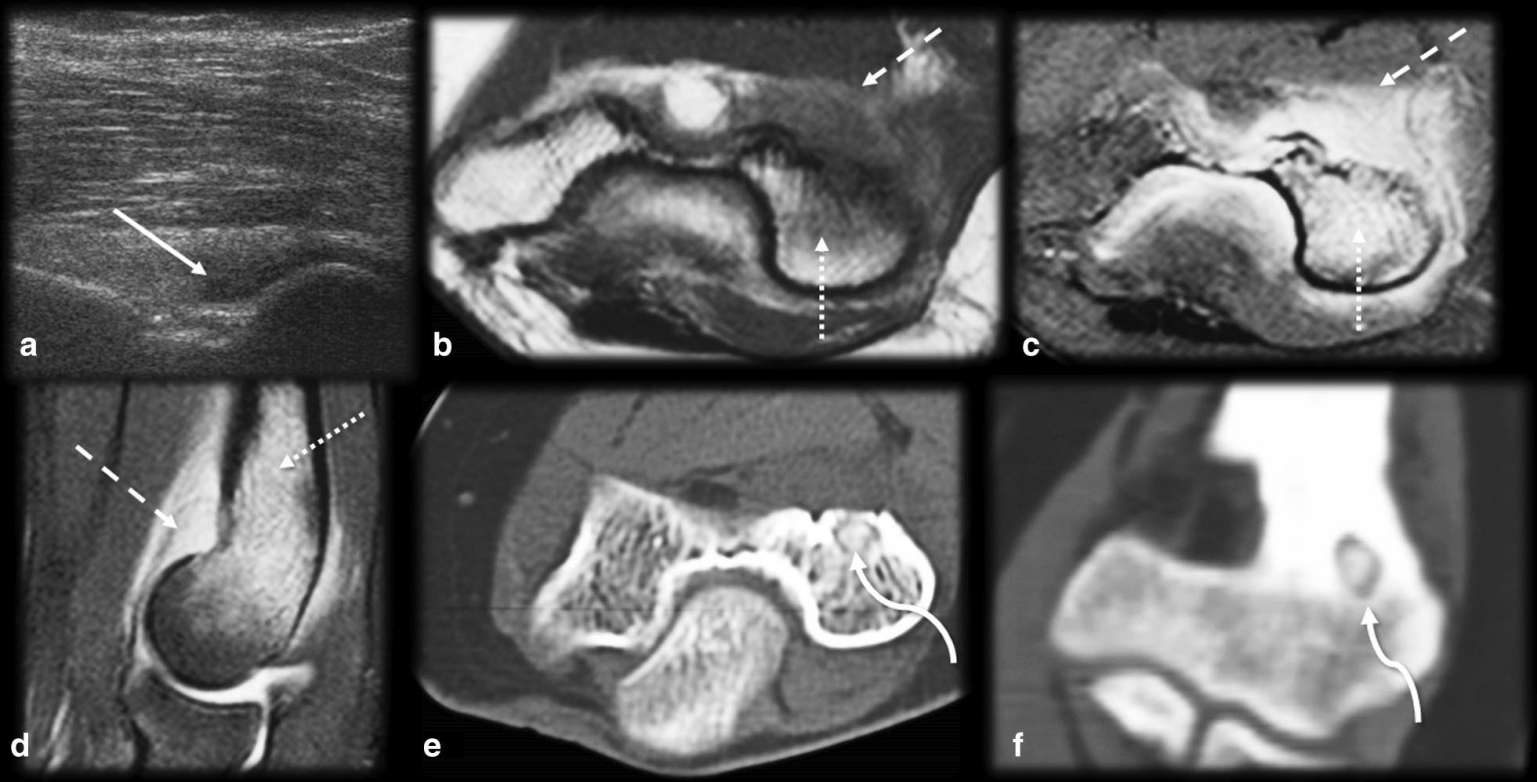
Osteoid osteoma mimicking synovitis on the elbow. Female, 20 years old, with elbow pain and edema for 4 months. Ultrasound in anterior sagittal view (**a**) at the coronoid fossa level shows joint effusion (arrow). MRI T1 (**b**) and T2FS (**c**,** d**) show bone marrow edema (dotted arrows) and synovitis (dashed arrows). Further investigation with a CT scan (**e**,** f**) revealed a mineralized nidus (curved arrow) in the cancellous bone of the medial humeral condyle

Intraarticularly located OO is uncommon, with an incidence of up to 16% [10]. The most common location is the hip (Fig. 4), and other joints, such as the ankle, elbow (Fig. 5), knee and wrist, are more rarely affected [14, 38]. Intraarticular prostaglandins promote lymphoproliferative synovitis, which leads to atypical clinical symptoms, such as arthritis, joint effusion, pain, stiffness and a high local temperature [9, 11, 14]. There is most often no nocturnal worsening and little improvement after NSAID treatment, so the condition is easily mistaken for inflammatory or infectious arthritis. The nidus is identified in only 28–50% of cases, and cortical thickening is reduced or absent in these cases since there is a small amount of periosteal apposition at the joint due to the absence of the cambium (internal) layer of the articular periosteum [4, 14]. The symptoms usually long precede the radiographic findings, and a delay in treatment may precipitate osteoarthritic changes in as many as 50% of cases [2, 6, 30, 39, 40].

OO may be localized within the cancellous bone, usually in atypical sites such as the metaphysis of long bones (the femoral neck is the most common location) and carpal/tarsal bones. The periosteal reaction and cortical thickening tend to be less marked in this type of OO than in typical OO [5, 14], and bone marrow edema is usually more intense, in which case MRI is more advantageous than CT [30].

**Fig. 6:**
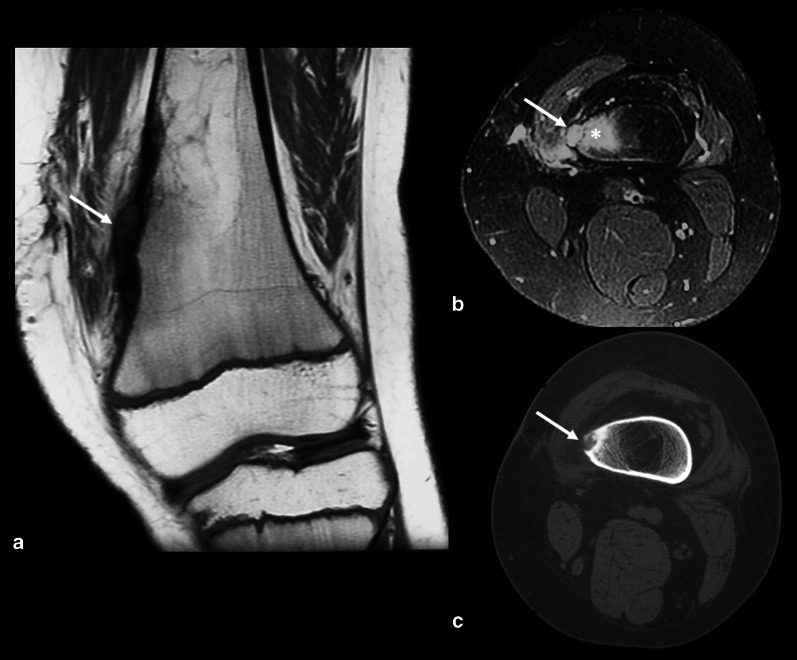
Osteoid osteoma near a growth plate. An 11–year-old male with a history of surgical removal of an OO on the distal metadiaphysis of the left femur. His symptoms persisted, and follow-up MRI (**a**, **b**) and CT (**c**) showed a residual nidus at the medial femoral margin represented by the intracortical nodule (arrows) and surrounding bone marrow edema (asterisk in **b**). In a, note that the affected side of the distal femur is longer than the lateral side, resulting in a femoral deformity and length discrepancy of the left lower limb

**Fig. 7:**
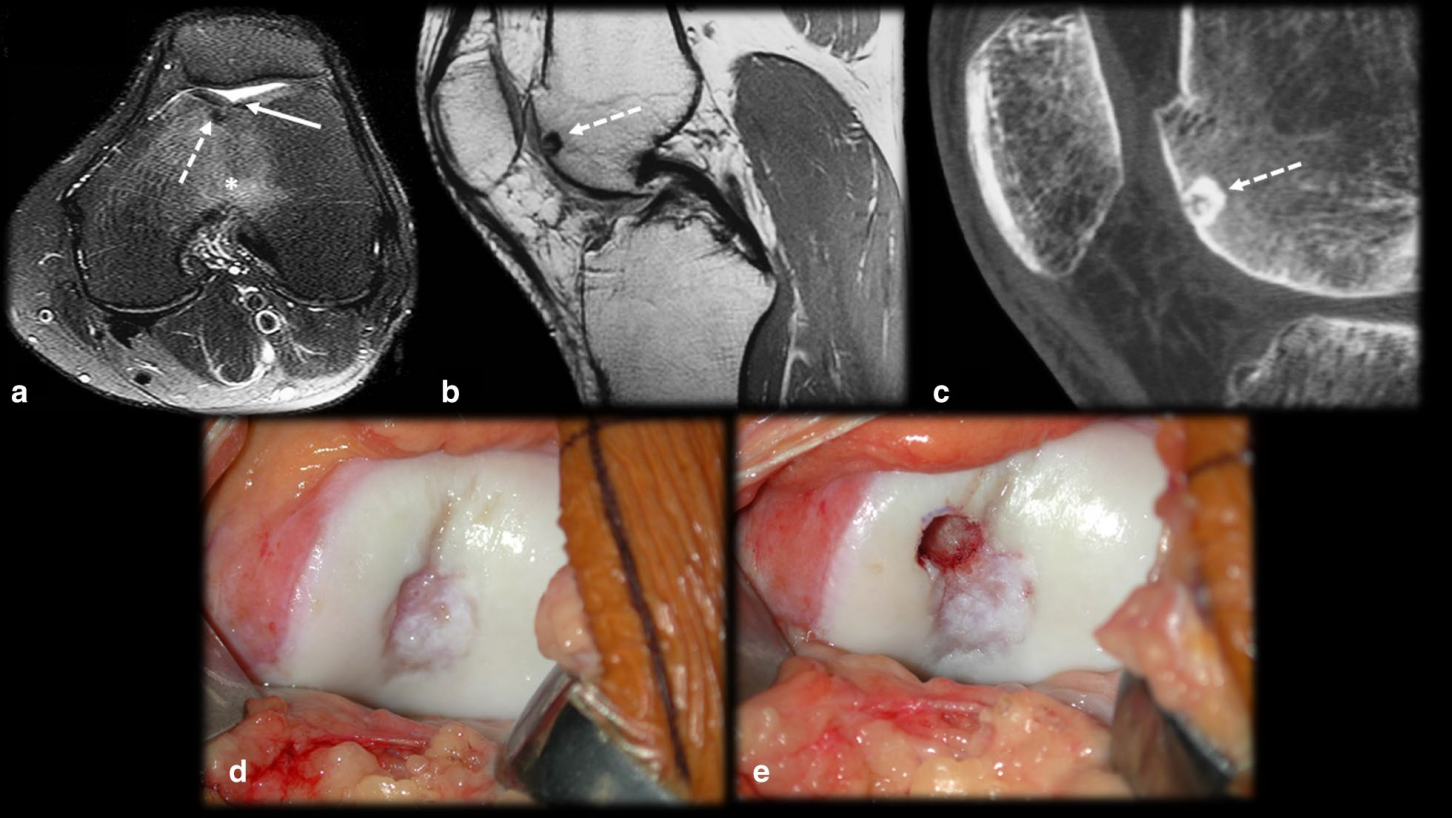
Subchondral osteoid osteoma mimicking trochlear chondromalacia. A 35-year-old male with anterior knee pain for 4 months. Axial T2 FS MR images (**a**) showed deep chondral erosion (arrow), subchondral edema (asterisk) and a small, low signal intensity foci that could be a nidus (dashed arrow). Sagittal T1 MRI (**b**) and CT (**c**) confirmed the diagnosis of the OO nidus (dashed arrow). Surgical images before (**d**) and after (**e**) resection

Epiphyseal OO cases are infrequent (less than 10%) and may be related to atypical features. Lesions close to the growth plate may cause bone length discrepancy, especially in very young children, and the affected limb is typically longer [3, 6, 8, 41] (Fig. 6). This type of OO may also cause premature fusion of the physis, angular deformity, joint contracture and muscle atrophy, resulting in growth disturbances [9]. Subchondral OO is even rarer and may be confused with chondromalacia (Fig. 7) due to the reactional changes in the subchondral bone being similar.

OO may also affect the distal extremities of the appendicular skeleton. Medullary OO is the most common type that occurs in carpal and tarsal bones, while all types may occur in the metacarpal, metatarsal and phalangeal bones. Medullary OO is usually accompanied by less cortical thickening than is typical OO and may induce bone expansion [41]. Since the bones of the hands and feet are small and close to each other, it may be difficult to locate the cause of inflammation, which may spread to adjacent bones, joints and soft tissues. Additionally, there may be prominent soft-tissue swelling, resembling infection or inflammatory arthritis [14, 41]. The nidus is very small and may be difficult to identify. When OO is located in the distal phalanx, it may also cause nail deformities, which are also confounding factors [41]. In addition, the clinical presentation may be unusual, with atypical pain or even the absence of pain, due to the absence of intralesional nerve fibers [39].

### Pitfalls, differential diagnoses and OO-mimicking lesions

Some pathologies may mimic OO due to there being similar imaging findings, such as cortical thickening, reactive sclerosis, small lytic lesions and bone marrow edema. In general, the presence of a large lesion, a medullary lesion, a small surrounding region of osteosclerosis, a periosteal reaction and bone marrow edema may help distinguish OO from mimicking lesions [20]. The main differential diagnoses are described below and summarized in Table 2.

**Table 2:** Painful OO’s differential diagnoses main imaging findings

Differential diagnoses	Key points
Osteomyelitis/intraosseous abscess	Uneven inner margin; irregularly shaped and eccentrically located sequestrum; usually intramedullary located and larger than 2.0 cm; does not enhance in its central portion; penumbra sign may be present
Fracture/stress reaction	Fracture line may be present; lack of a nidus; Follow-up imaging can be helpful in doubtful cases because the bone marrow edema regress over time and the fracture consolidates
Osteoblastoma	Larger than 2.0 cm; less painful; fewer inflammatory changes and reactive sclerosis; smaller response to salicylates; grow progressively; malignant potential and may be associated with other tumors
Glomus tumor	Well-defined nodule in the nail bed; no thickening of the rest of the nail bed or matrix; may exhibit well-defined remodeling of the dorsal cortical of the distal phalanx
Chondroblastoma	Epiphyseal intramedullary location; lobulated contours; larger dimensions; chondral calcifications and signal intensity

**Fig. 8:**
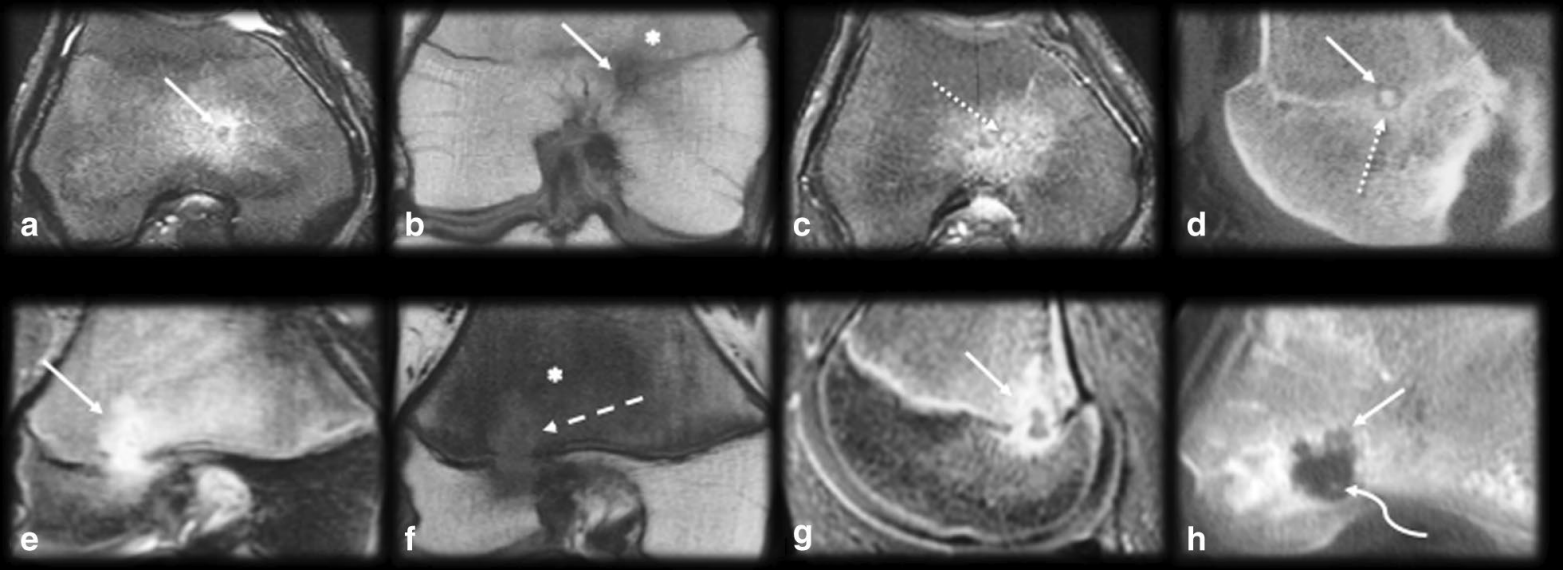
Osteoid osteoma versus osteomyelitis. (**a**–**d**) Male, 15 years old, presenting with knee pain for 2 months. MRI T2 FS (**a**), T1 (**b**), T1 FSGD (**c**) and CT (**d**) showed a nidus with smooth margins (arrows), a central mineralized portion (dashed arrows), homogeneous gadolinium enhancement (dotted arrows) and hazy T1 bone marrow edema around the lesion (asterisk). (**e**–**h**) Male, 13 years old, presenting with knee pain for 6 weeks. MRI T2 FS (**e**), T1 (**f**), T1 FS GD (**g**) and CT (**h**) showed a bone abscess with irregular margins and peripheral enhancement (arrows). Note there was mild bone marrow edema (asterisk), a positive penumbra sign (dashed arrow in **f**) and a small peripheral bone sequestrum (curved arrow in **h**)

*Osteomyelitis/intraosseous abscess* (Fig. 8): A small osseous abscess with internal bone sequestrum may resemble the mineralized nidus of OO and vice versa, especially on plain radiographs. However, some features allow the nidus to be differentiated from osseous abscesses in sectional studies. The inner margin of an abscess is usually uneven, and the sequestrum is irregularly shaped and eccentrically positioned; in contrast, in OO cases, the margins are smooth, and nidus mineralization is regular and central [9, 14]. Moreover, an abscess is usually larger than 1.0–2.0 cm and does not enhance in its central portion (since it consists of bone necrosis and pus), while OO lesions show strong enhancement of the nidus, except for the mineralized portion [14, 30]. Dynamic MR images may also be helpful since the nidus presents early arterial enhancement [16]. The penumbra sign (Fig. 7), characterized by a high signal intensity halo on T1WIs around the lesion, is nonspecific but indicates the possibility of infectious diseases [42].

**Fig. 9:**
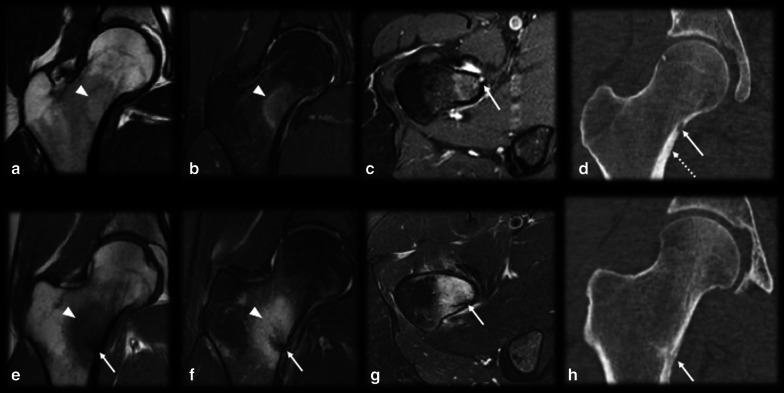
Osteoid osteoma versus calcar femorale stress fracture. (**a**–**d**) Male, 35 years old, presenting with hip pain for 3 months. MRI T1 (**a**) and T2 FS (**b**) showed bone marrow edema on the femoral neck (arrowhead), T1 FS GD (**c**) and CT (**d**) showed a small nonmineralized nidus (arrows) with gadolinium enhancement (**c**) and mild cortical thickening (dotted arrow in d). (**e**–**h**) Male, 33-year-old runner, presenting with hip pain for 2 weeks, which worsened during running workouts. MRI T1 (**e**) and T2 FS (**f**, **g**) showed bone marrow edema on the lower femoral neck (arrowhead) and a cortical fracture line (arrows), which was also seen on the CT scan (**h**)

**Fig. 10:**
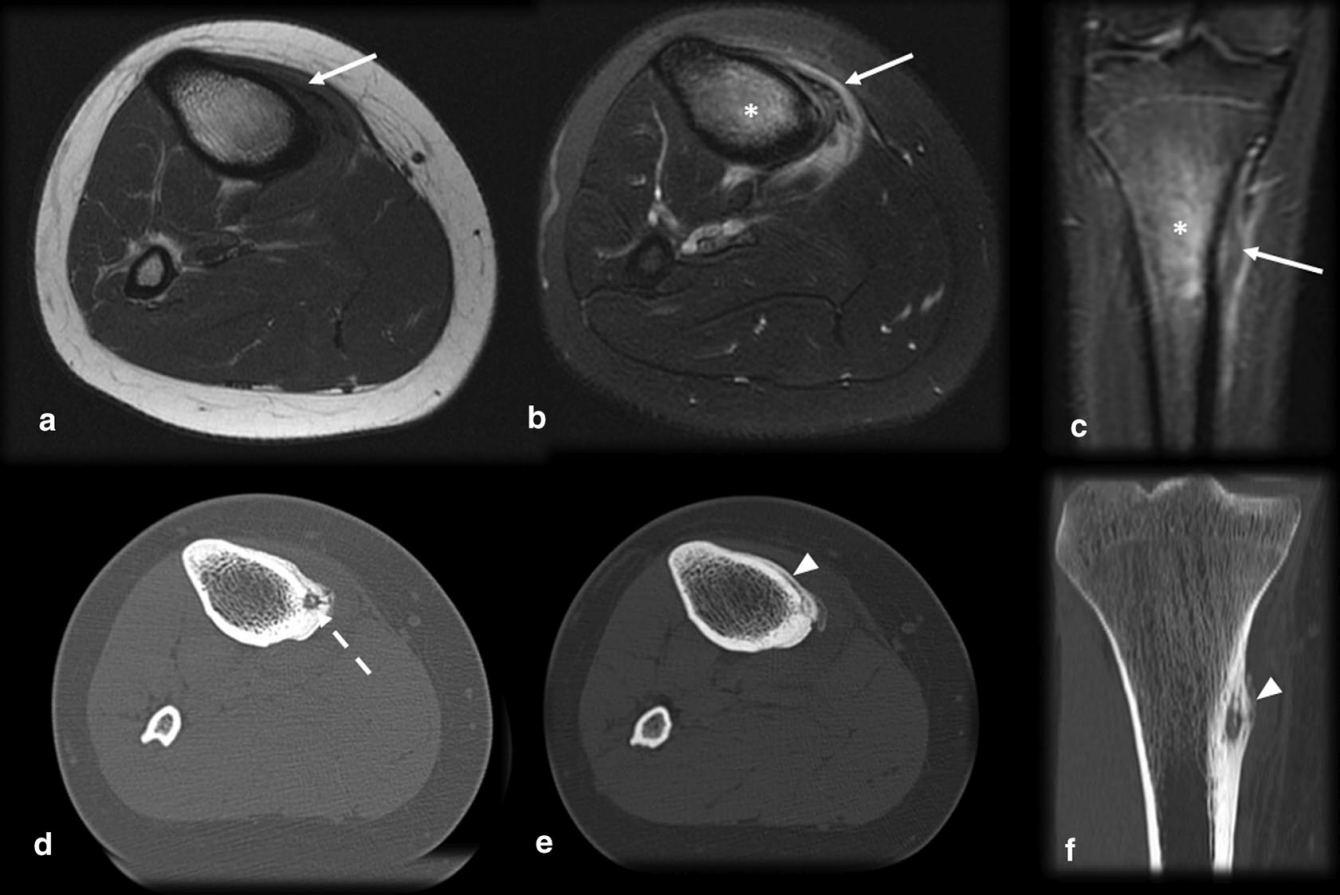
Osteoid osteoma versus tibial stress syndrome. A 15-year-old male soccer player with posteromedial tibial pain for 3 months that worsened while training and upon palpation of the upper posteromedial tibia. MRI axial T1 (**a**), axial (**b**) and coronal (**c**) T2 FS showed cortical thickening, periosteal reaction, pes anserinus tendon edema (arrows) and bone marrow edema (asterisk), mimicking a stress syndrome. Further investigation with a CT scan (**d**,** e**,** f**) demonstrated a nidus (dashed arrow), causing this corticoperiosteal reaction (arrowhead) and confirming OO

**Fig. 11:**
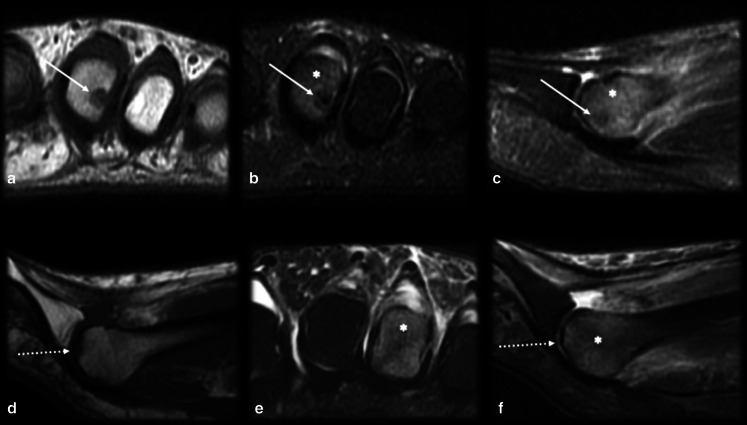
Osteoid osteoma versus subchondral fracture. (**a**–**c**) Female, 32 years old, presenting with metatarsalgia for 2 months. T1 coronal (**a**) and T2 FS coronal (**b**) and sagittal (**c**) MRI showed a mineralized nidus (arrows) with reactional bone marrow and adjacent edema (asterisk). (**d**–**f**) Female, 33 years old, presenting with metatarsalgia for 3 weeks. T1 sagittal (**a**) and T2 FS coronal (**b**) and sagittal (**c**) MRI showed a subchondral fracture (dotted arrows) with bone marrow edema (asterisk)

*Fracture/stress reaction* (Figs. 9, 10, 11): In young patients who practice physical activities, this differential diagnosis may be problematic since both fractures and OO frequently occur in the femoral neck region (Fig. 9) and tibia diaphysis (Fig. 10). In stress fracture cases, periosteal reactions, the fracture line and bone marrow edema can be visualized. In OO cases, although there may be edema and periosteal reactions, the unequivocal nidus characterization and absence of a cortical fracture confirm the presence of OO [14, 43]. However, if the diagnosis remains uncertain, a CT scan should be performed to detect either cortical discontinuity or the nidus. Follow-up imaging is also helpful since fractures/stress reactions consolidate and bone marrow edema cases regress over time [14]. Depending on the location of OO, subchondral fractures might also have a similar presentation (Fig. 11).

*Osteoblastoma*: Although some authors consider OO and osteoblastoma as spectra of the same pathology, most papers and the WHO classify these tumors as separate entities [44]. The two lesions, although very similar, present important clinical and radiological differences: osteoblastomas are larger, typically measuring more than 2.0 cm; are less painful; have a smaller response to salicylates; grow progressively; have the potential to be malignant; may be associated with other tumors; lead to fewer inflammatory changes; and less often lead to reactive sclerosis [14, 45, 46].

**Fig. 12:**
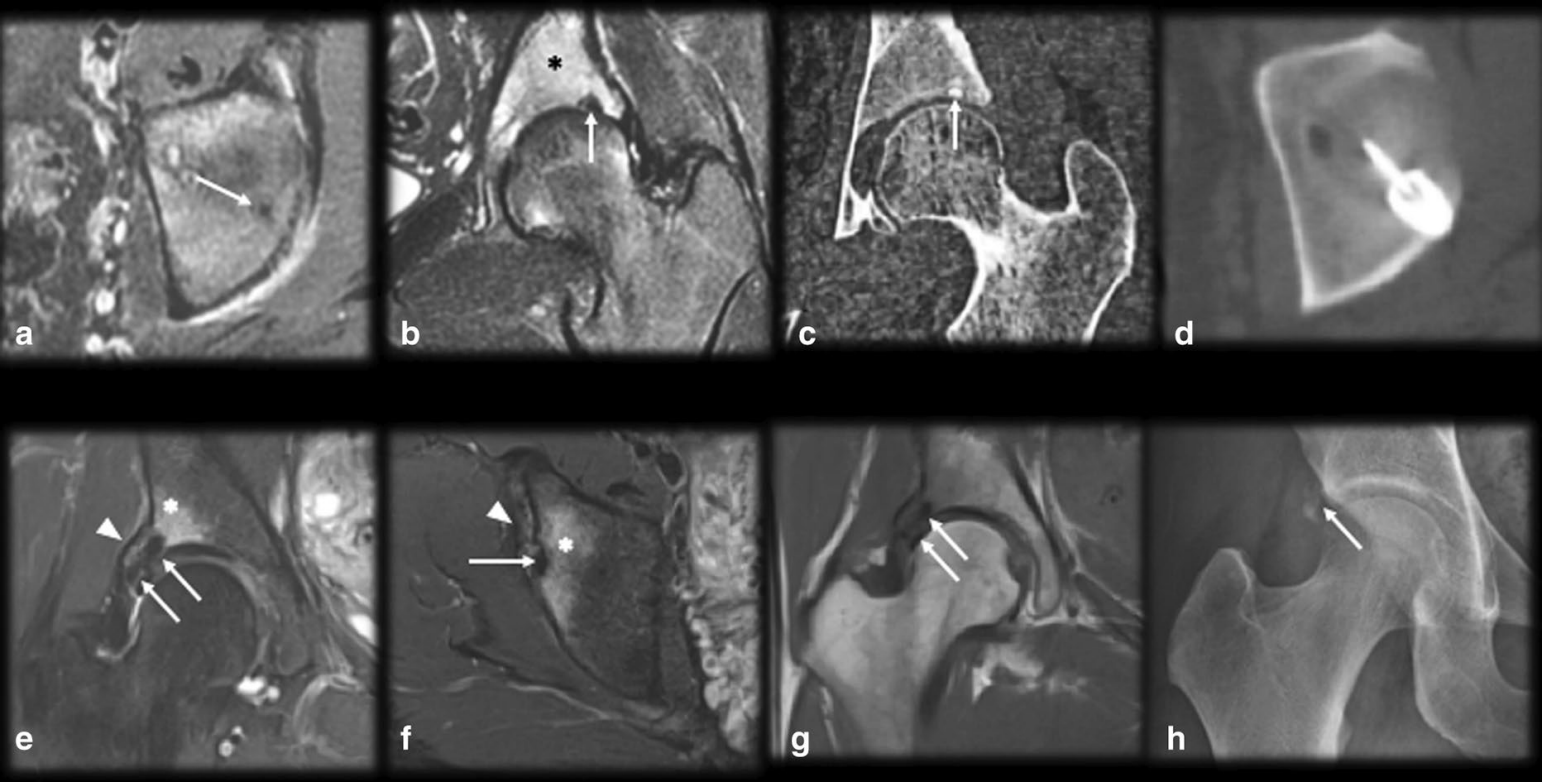
Osteoid osteoma versus crystal deposition disease. (**a**–**d**) Male, 19 years old, presenting with groin pain for one year. Axial (**a**) and coronal (**b**) T2 FS MRI and CT (**c**) showed a small mineralized nidus (arrows) and reactional bone marrow edema (black asterisk). Percutaneous CT-guided drill excision was performed (**d**). (**e–h**) Female, 36 years old, presenting with hip pain for 5 months. Coronal (**e**) and axial (**f**) T2 FS and coronal T1 (**g**) MRI showed calcifications (arrows) close to the indirect head of rectus femoris (arrowheads), which were better characterized on the plain radiograph (**h**), with reactional bone marrow edema (asterisk)

**Fig. 13:**
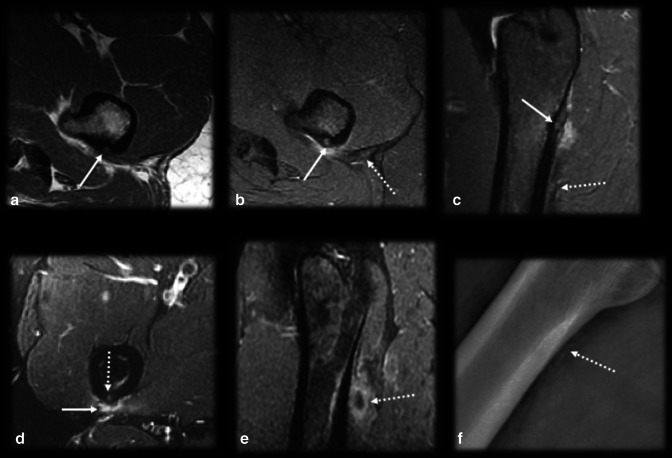
Osteoid osteoma versus crystal deposition disease [2]. (**a**–**c**) Male, 30 years old, presenting with posterior thigh pain for 3 months. Axial T1 (**a**) and axial (**b**) and sagittal (**c**) T2 FS MRI demonstrated a cortical nidus (arrows) and reactional bone marrow edema close to the gluteus tuberosity. Note that the gluteus maximus tendon (dotted arrow) insertion is below the nidus. (**d**–**f**) Female, 38 years old, presenting with very intense posterior thigh pain for 2 days. T2 FS axial and coronal MRI (**d**,** e**) and plain radiograph (**f**) showed corticoperiosteal and bone marrow edema in the right gluteus maximus tendon (arrows) insertion and some calcifications (dotted arrows)

*Crystal deposition disease* (Figs. 12, 13): Crystal deposition disease can occur at any site, such as the tendons, ligaments, fibrocartilage or joint capsule, and may complicate the differential diagnosis for OO when there is intraosseous migration leading to cortical remodeling and bone marrow edema (Figs. 12, 13). Age should be considered for the differentiation between these entities since OO affects mainly younger patients, and the microcrystal deposition usually affects an older age group [47]; however, there is considerable overlap around the 4th decade of life, especially regarding hydroxyapatite deposition. Ultrasound and CT are useful for visualizing the extension and location of the calcifications, allowing the identification of extraosseous calcific foci associated with crystal deposition.

**Fig. 14:**
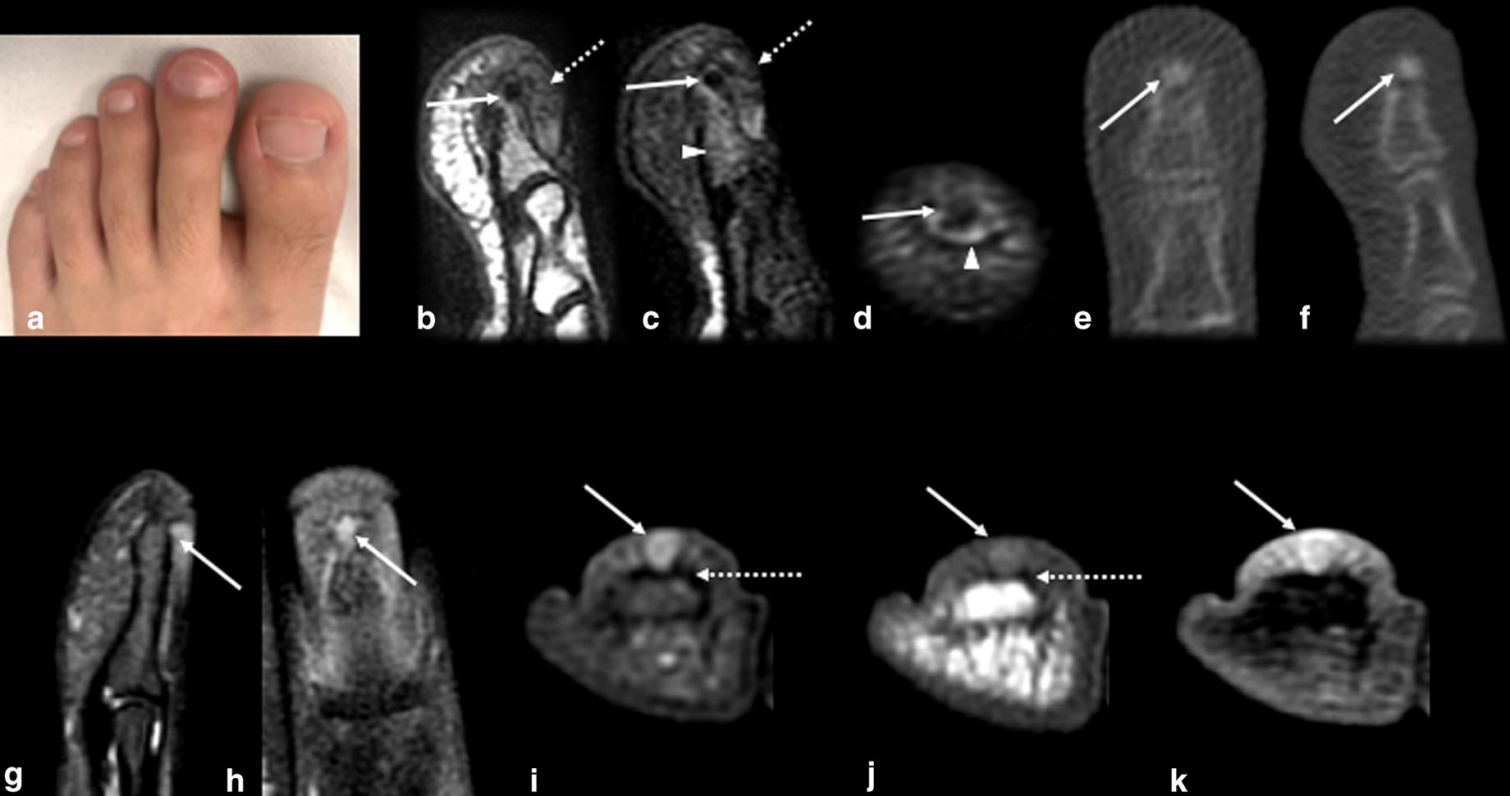
Osteoid osteoma versus glomus tumor. (**a**–**f**) Male, 19 years old, presenting with second toe clubbing and night pain (**a**). Sagittal T1 (**b**) and sagittal (**c**) and axial (**d**) T2FS MR images showed diffuse nail bed thickening (dotted arrows), with no defined nodule, as well as a low signal cortical/juxtacortical nodule at the distal phalanx (arrows), accompanied by bone marrow edema (arrowhead). A CT scan (**e**,** f**) was performed, and the findings revealed a sclerotic nodule corresponding to an OO nidus (arrows), with minimal reactional surrounding sclerosis. (**g**–**k**) Female, 53 years old, presenting with pain on the 4th finger that radiated to the forearm. Sagittal, coronal, axial T2 FS, axial T1 and T1 FS GD MR images depicted a subungual nodular well-defined lesion (arrow) with remodeling of the subjacent phalanx cortex (dotted arrow) and homogeneous enhancement after gadolinium injection (**k**). Note that diffuse thickening of the nail bed or phalanx sclerosis was absent.

*Glomus tumor* (Fig. 14): OO of the distal phalanx is often associated with an atypical clinical picture, with little to no pain, single-digit clubbing and diffuse thickening of the nail bed, with a high T2WI signal intensity and gadolinium enhancement, which can lead to the erroneous diagnosis of a glomus tumor, especially if MRI is the only available imaging modality [48–50]. However, glomus tumors are well-defined nodules in the nail bed, with no thickening of the rest of the nail bed or matrix and may exhibit remodeling of the dorsal cortical of the distal phalanx [51] (Fig. 14). Moreover, single-digit clubbing is relatively rare, and the possibility of primary bone neoplasm should always be investigated, with enchondroma and OO representing the most common types of neoplasm with this manifestation [48–50].

*Aggressive bone lesions*: Most aggressive bone lesions have very different imaging patterns than does OO, as they are characterized by the replacement of bone marrow with a markedly low T1 signal intensity, leading to a generally well-demarcated transition with preserved bone marrow, and they may also exhibit cortical rupture and extracortical involvement. In contrast, the pattern of edema that occurs in OO cases is characterized by a gray, hazy and ill-defined T1 intermediate signal intensity, with no substitution of bone marrow.

**Fig. 15:**
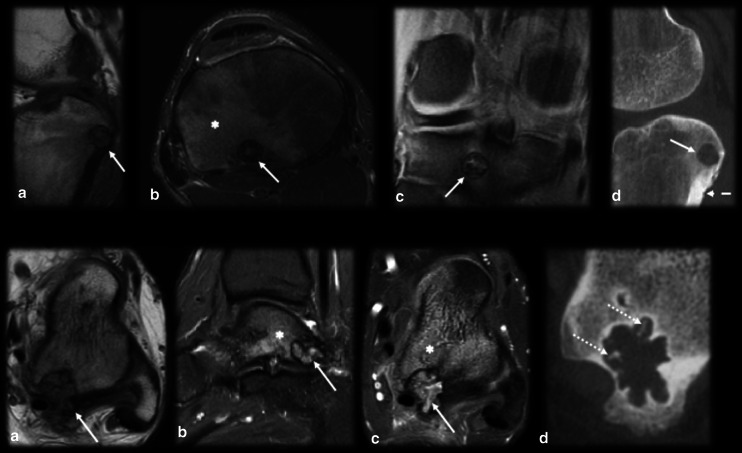
Osteoid osteoma versus chondroblastoma. (**a**–**d**) Male, 29 years old, presenting with knee pain for 3 months. MRI sagittal T1 (**a**), axial and coronal T2 FS (**b**,** c**) and CT (**d**) showed a round and central mineralized nidus (arrows) with reactional bone marrow edema (asterisk) and cortical thickening (dashed arrow in d). (**e**–**h**) Male, 24 years old, presenting with ankle pain for 9 months. MRI axial T1 (**e**), sagittal T2 (**f**), axial T1 FS GD (**g**) and CT (**h**) showed a large and lobulated bone lesion (arrows) with peripheral arciform calcifications (dotted arrows), internal enhancement (**c**) and reactional bone marrow edema (asterisk)

*Chondroblastoma* (Fig. 15): Chondroblastomas are rare and painful benign bone neoplasms that are generally smaller than 4.0 cm. They predominantly occur in epiphyses or apophyses of immature bones; they are most prevalent in the femur, followed by the humerus and tibia; and they are predominant in males. The lesion is lytic, central or eccentric intramedullary, with well-defined limits, a thin sclerotic halo, a high T2 signal intensity and gadolinium enhancement. Central chondroid-pattern calcifications may be present in approximately 30–40% of cases. This condition is associated with inflammatory changes and is sometimes accompanied by synovitis and surrounding soft-tissue edema [14]. Epiphyseal and medullary localization, lobulated contours, chondral-like calcifications and larger dimensions may aid in the differentiation of chondroblastoma from OO, which is usually smaller and located on cortical bone and on the diaphysis; however, small and mineralized chondroblastoma may be indistinguishable from OO [14].

**Fig. 16:**
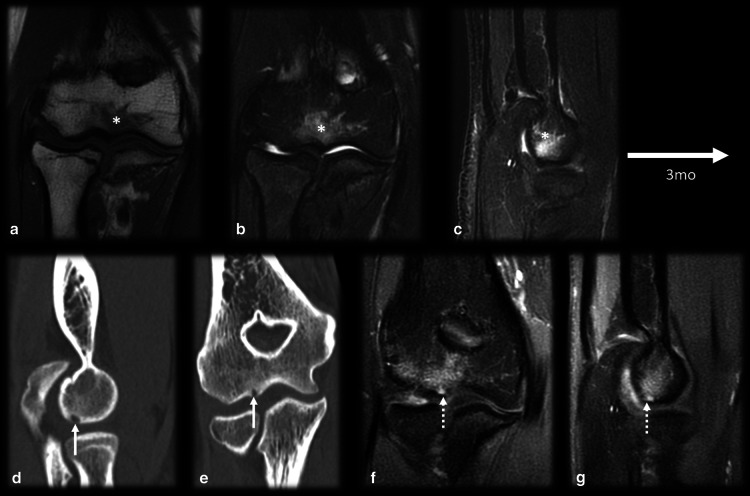
Osteoid osteoma mimicking bone marrow contusion. Male, 16 years old, presenting with right elbow and arm pain for 2 days after falling during physical activity at school. First, the MRI T1 (**a**) and T2 FS (**b**,** c**) findings were interpreted to indicate contusion bone marrow edema (asterisks) without a fracture. However, the pain persisted for more than 3 months. Follow-up CT (**d**,** e**) and MRI (**f**,** g**) scans revealed small subchondral OO with a lytic nonmineralized nidus (arrows) and enhancement (dotted arrows) on the T1 FS GD MRI scan (**f**,** g**)

**Fig. 17:**
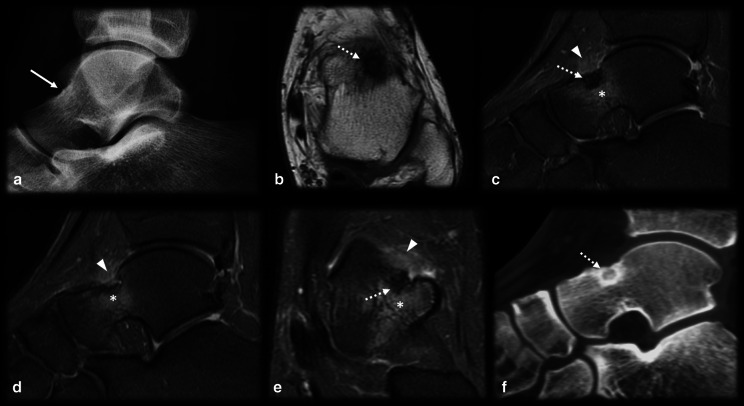
Osteoid osteoma mimicking anterior impingement. A 29-year-old male presenting with anterior ankle pain for 4 months. CR (**a**) showed mild sclerosis on the dorsal talar neck (arrow). T1 (**b**), T2 (**c**,** d**,** e**) and CT (**f**) showed a mineralized nidus (dotted arrows) with reactional synovitis (arrowhead), bone marrow edema (asterisk) and sclerosis

**Fig. 18:**
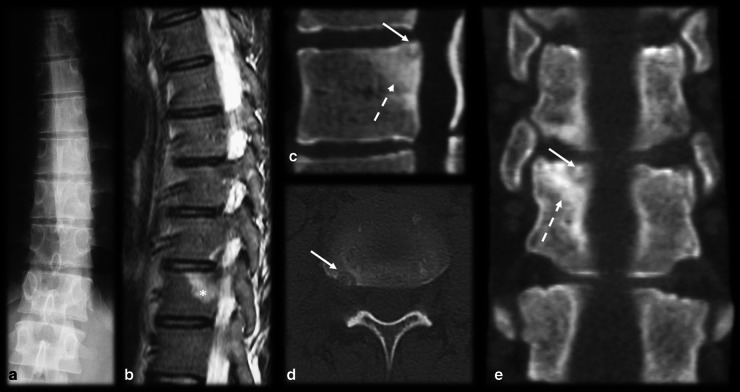
Osteoid osteoma mimicking enthesitis. Male, 20 years old, presenting with back pain for six weeks. CR (**a**) showed left scoliosis with no identifiable bone lesions. Sagittal T2 FS MRI (**b**) revealed bone marrow edema on the posterosuperior corner of the vertebral body (asterisk), which raised the suspicion for enthesitis. The CT scan (**c**,** d**,** e**) showed a small and mineralized nidus on the concave side of the region of scoliosis (arrow) with reactional bone sclerosis (dashed arrow)

*Miscellaneous* (Figs. 16, 17, 18): OO can resemble pathologies other than those previously mentioned, such as contusional bone marrow edema (Fig. 16), impingements (Fig. 17), enthesitis (Fig. 18), compensatory hypertrophy of the pedicle, intracortical hemangioma, osteochondroses, cortical desmoid, fibrous dysplasia and eosinophilic granuloma [4, 6, 14].

## Conclusion

The clinical and radiological profile of OO can be very similar to that of other pathologies. The atypical forms of presentation, differential diagnoses and active nidus characteristics need to be investigated further especially in volumetric studies, including CT, to avoid errors and delays in the diagnosis, thereby leading to the selection of appropriate treatments for and good prognosis in patients**.**

## Data Availability

Not applicable.

## Abbreviations

CR: Conventional radiography
CT: Computed tomography
FS: Fat saturated
GD: Gadolinium
MRI: Magnetic resonance imaging
OO: Osteoid osteoma
T1WI: T1 weighted image
T2WI: T2 weighted image
